# The antibody response to *Plasmodium falciparum *Merozoite Surface Protein 4: comparative assessment of specificity and growth inhibitory antibody activity to infection-acquired and immunization-induced epitopes

**DOI:** 10.1186/1475-2875-10-266

**Published:** 2011-09-16

**Authors:** Harini D de Silva, Suha Saleh, Svetozar Kovacevic, Lina Wang, Casilda G Black, Magdalena Plebanski, Ross L Coppel

**Affiliations:** 1 Department of Microbiology, Monash University, Clayton, Victoria, Australia; 2 Department of Immunology, Monash University, Prahran, Victoria, Australia

## Abstract

**Background:**

Malaria remains a global public health challenge. It is widely believed that an effective vaccine against malaria will need to incorporate multiple antigens from the various stages of the parasite's complex life cycle. *Plasmodium falciparum *Merozoite Surface Protein 4 (MSP4) is a vaccine candidate that has been selected for development for inclusion in an asexual stage subunit vaccine against malaria.

**Methods:**

Nine monoclonal antibodies (Mabs) were produced against *Escherichia coli*-expressed recombinant MSP4 protein and characterized. These Mabs were used to develop an MSP4-specific competition ELISA to test the binding specificity of antibodies present in sera from naturally *P. falciparum*-infected individuals from a malaria endemic region of Vietnam. The Mabs were also tested for their capacity to induce *P. falciparum *growth inhibition *in vitro *and compared against polyclonal rabbit serum raised against recombinant MSP4

**Results:**

All Mabs reacted with native parasite protein and collectively recognized at least six epitopes. Four of these Mabs recognize reduction-sensitive epitopes within the epidermal growth factor-like domain found near the C-terminus of MSP4. These sera were shown to contain antibodies capable of inhibiting the binding of the six Mabs indicating infection-acquired responses to the six different epitopes of MSP4. All of the six epitopes were readily recognized by human immune sera. Competition ELISA titres varied from 20 to 640, reflecting heterogeneity in the intensity of the humoral response against the protein among different individuals. The IgG responses during acute and convalescent phases of infection were higher to epitopes in the central region than to other parts of MSP4. Immunization with full length MSP4 in Freund's adjuvant induced rabbit polyclonal antisera able to inhibit parasite growth *in vitro *in a manner proportionate to the antibody titre. By contrast, polyclonal antisera raised to individual recombinant fragments rMSP4A, rMSP4B, rMSP4C and rMSP4D gave negligible inhibition. Similarly, murine Mabs alone or in combination did not inhibit parasite growth.

**Conclusions:**

The panel of MSP4-specific Mabs produced were found to recognize six distinct epitopes that are also targeted by human antibodies during natural malaria infection. Antibodies directed to more than three epitope regions spread across MSP4 are likely to be required for *P. falciparum *growth inhibition *in vitro*.

## Background

Malaria infections of humans, particularly that due to *Plasmodium falciparum *continues to be a major cause of morbidity and mortality in tropical countries. There is an urgent need for the development of efficacious control measures, one component of which could be a safe, effective and affordable malaria vaccine against *P. falciparum*. It is widely believed that any such vaccine will need to incorporate multiple antigens from the various stages of the parasite's complex life cycle [1].

The surface of the asexual stage merozoite form of *P. falciparum *is composed of a number of proteins that are the targets of immune attack by antibodies. One of these proteins is Merozoite Surface Protein 4 (MSP4), a relatively abundant glycosylphosphatidylinositol-anchored protein that contains a single epidermal growth factor (EGF)-like domain adjacent to the carboxyl terminus of the protein [2,3]. Although the function of MSP4 is not known, the *msp4 *gene is refractory to genetic deletion and it is thus thought to be essential for parasite replication in *in vitro *culture and presumably also in the human bloodstream [4]. Several features of MSP4 make it an attractive vaccine candidate. Firstly, MSP4 is exposed on the merozoite surface making it available for antibody binding and anti-MSP4 antibodies are readily detected in people living in malaria endemic regions [5,6] suggesting a possible role for these antibodies in human immunity to malaria. Secondly, MSP4 shows a high degree of conservation among *P. falciparum *isolates [7-9] minimizing the possibility of immune evasion secondary to strain-specific antibody responses. Thirdly, immunization of mice with recombinant *Plasmodium yoelii *MSP4/5, a homologue of both MSP4 and the related antigen MSP5, protects mice against lethal parasite challenge [10,11]. Protection is enhanced when MSP4/5 is immunized in combination with *P. yoelii *MSP1_19 _[12] suggesting that it would be an attractive addition to a multi-antigen vaccine containing MSP1_19_.

A panel of nine anti-MSP4 monoclonal antibodies (Mabs) that recognize distinct epitopes of the antigen were produced and characterized. These antibodies were tested in a competition enzyme-linked immunosorbent assay (ELISA) against human immune sera collected from *P. falciparum*-infected subjects to analyse the binding characteristics of anti-MSP4 antibodies induced by natural infection. The ability of polyclonal and monoclonal anti-MSP4 antibodies to inhibit parasite growth *in vitro *were also assessed in this study.

## Methods

### Production of antigens

#### Parasite proteins

*Plasmodium falciparum *isolate 3D7 was cultured *in vitro *as previously described [3] and total parasite protein preparations were obtained by saponin lysis of parasites as previously described [5].

#### Recombinant proteins

**Figure 1:**
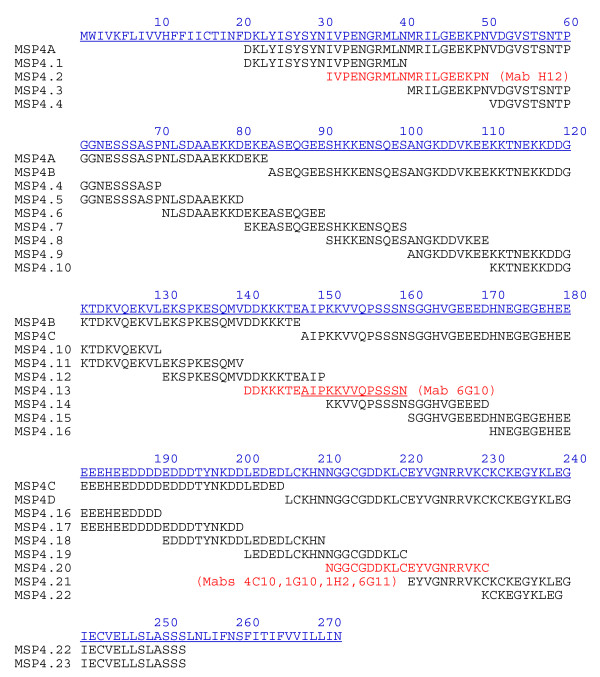
**Location of Mab epitopes within the MSP4 amino acid sequence**. Full amino acid sequence of MSP4 (blue) is aligned with the sequences corresponding to four recombinant fragments (MSP4A: amino acids D_21_-E_83_; MSP4B: A_84_-E_147_: MSP4C: A_148_-D_203_; MSP4D: L_204_-S_248_), and 23 synthetic peptides (MSP4.1 - MSP4.23). The epitopes for a number of Mabs are indicated in red within several of the peptides: Mab H12 (MSP4.2), Mab 6G10 (MSP4.13), and Mabs 4C10, 1G10, 1H2, 6G11 (MSP4.20).

Full-length MSP4 consisting of amino acid residues 21-248 was expressed in *E. coli *as a recombinant hexahistidine tagged protein (rMSP4) or as a GST fusion protein (rMSP4^GST^) as described [5] and also expressed in *Saccharomyces cerevisiae *(rMSP4^Sc^) as described [13]. The protein, rMSP4, was used to immunize mice and in ELISA as a target antigen. In order to map the epitopes corresponding to different regions of the MSP4 protein, four previously described glutathione S-transferase (GST) recombinant fusion proteins designated rMSP4A, rMSP4B, rMSP4C and rMSP4D were produced [5]. Each fragment represents approximately one quarter of the mature MSP4 protein (MSP4A corresponding to amino acids 21-83; MSP4B to amino acids 84-147; MSP4C to amino acids 148-203 and MSP4D to amino acids 204-248) (Figure 1). The rMSP1_19 _was a kind gift from Paul Gilson (WEHI, Melbourne).

#### MSP4 synthetic peptides

Overlapping peptides spanning the entire length of the MSP4 molecule were synthesized by Auspep Pty Ltd. (Melbourne, Australia). All peptides were 20 amino acids in length and each adjacent peptide overlapped by 10 amino acids (Figure 1).

#### Human and animal polyclonal antibodies

Human immune sera were collected from residents living in Khanh Hoa province in south central Vietnam. Initial blood samples were obtained from volunteers in June 1994 (designated T_0_). These individuals were thoroughly treated with quinine sulphate, doxycycline cyclate and primaquine phosphate then monitored for a period of 6 months [6]. Additional blood samples (T_1_) were collected from those individuals who had positive smears during the 6-month period. They were treated with mefloquine (15 mg/kg) and a third sample (T_28_) was collected 28 days later. No volunteers had recurrent parasitaemia during the 28 days of follow-up after mefloquine treatment. A panel of individual serum samples were obtained from 30 healthy Australian blood donors and pooled for use as negative control sera. None of these donors had a history of exposure to malaria.

Rabbit antisera to rMSP4, rMSP4A, rMSP4B, rMSP4C, rMSP4D, rMSP4^GST^, rMSP4^Sc ^and MSP1_19 _were produced in New Zealand White rabbits using Freund's adjuvants as previously described [5]. Rabbit anti-AMA-1 as purified IgG was a kind gift from Carole Long (NIH, USA).

#### Production of Mabs

Briefly, BALB/c mice were immunized intraperitoneally with 25 μg of rMSP4 protein in Freund's complete adjuvant, followed by two further injections of 25 μg protein in Freund's incomplete adjuvant 21 and 34 days later. Serum was tested after the final immunization by ELISA for the presence of anti-MSP4 antibodies. A final boost of 25 μg protein in PBS was given and three days later the mice were killed and spleen cells fused with SP2 myeloma cells and hybridomas selected as described previously [14]. Anti-MSP4 antibodies were screened for reactivity to the immunizing antigen by ELISA. Reactivity to native protein was also assessed by immunoblotting 3D7 extracts and by indirect immunofluorescence assay (IFA) to fixed merozoites [15]. Monoclonality was ensured by limiting dilution twice and further testing as described above. Selected clones were expanded by growth in RPMI medium containing 10% (v/v) bovine calf serum. The IgG subclass of each Mab was determined by ELISA using an isotype determination kit (Sigma Poole, UK). Mabs were purified from hybridoma supernatant using G-Sepharose 4 protein purification columns (Pharmacia Biotech, Victoria, Australia) and biotinylation of Mabs was carried out using Sulfo-NHS-Biotin (Pierce Biotechnology) according to the manufacturer's recommendations.

#### Immunoblotting

Parasite extracts or recombinant MSP4 were resolved on 12% (v/v) polyacrylamide gels by SDS-PAGE under non-reduced, reduced, or reduced and alkylated conditions and transferred to PVDF membrane for immunoblotting as described previously [5]. Primary anti-MSP4 Mab binding was detected with anti-mouse immunoglobulin conjugated to horseradish peroxidase (Silenus Laboratories, Melbourne, Australia) and developed using the Renaissance Chemiluminescence Reagent (NEN Life Science Products, Boston, Mass., USA).

#### Indirect ELISA

All procedures were carried out as described previously [5] with minor modifications. Briefly, 96 well microtiter plates (Immulon 2; Dynatech Laboratories, Chantilly Va.) were coated with recombinant MSP4 (1 μg/mL) or synthetic peptides (10 μg/mL) at 4°C overnight, washed with PBS/0.5% (v/v) Tween 20 (PBST) and blocked with 5% (w/v) skim milk powder in PBST. Mabs were added in duplicate wells. Primary antibody was detected with alkaline-phosphatase conjugated goat anti-mouse immunoglobulin (Silenus Laboratories, Melbourne, Australia) followed by development with *p*-nitrophenyl phosphate (Sigma). The optical density (OD) was read at 405 nm. Each sample was tested against PBS or RPMI medium as a background control. Specific reactivity against MSP4 protein was obtained by subtracting the average OD of the control wells from the average OD obtained for wells coated with antigen.

#### Competition ELISA with biotinylated Mabs

Microtiter plates were coated with rMSP4D and other steps performed as described previously [16]. Briefly, unlabelled Mab was added and incubated for one hour at 37°C followed by washing and addition of the biotinylated Mab at a concentration predetermined by titration and incubated as above. After washing, Streptavidin-HRP conjugate (50 μL/well; Pierce Biotechnology) was added and incubated as above, followed by development with *o*-phenylenediamine substrate (75 μL/well; Sigma). The colour reaction was stopped by addition of 50 μL of 1 M sulfuric acid and the absorbance was read at 490 nm. The degree of inhibition for each antibody was calculated using the following equation: I (%) = [(OD_L _- OD_U+L_)/ OD_L_] × 100, where I = inhibition (%); OD_L _= OD_405 _of the labelled antibody alone and OD_U+L _= OD_405 _of the labelled antibody where unlabelled antibody had been added.

#### Competition ELISA with human serum

Microtiter plates were coated with rMSP4B, rMSP4C or rMSP4D, blocked as above and antigen reacted with serial two-fold dilutions of human sera as described previously [17]. After washing, 50 μL/well of each anti-MSP4 Mab was added and incubated for two hours at 37°C. All other steps were as described for indirect ELISA. Serum samples were tested against PBS as a negative control. The competition titres were defined as the dilutions of the test sera that resulted in equal or higher OD_405 _values as the mean plus the standard deviations (2SD) of the OD_405 _values of the 30 negative control sera. Sera with a titre ≥ 20 were considered positive. The percentage inhibition of each serum at 1/10 dilution was calculated using the following equation: I (%) = [(OD_IFS _- OD_TS_)/ OD_IFS_] × 100, where I = inhibition (%); OD_IFS _= OD_405 _of the inhibitor-free sample and OD_TS _= OD_405 _of the test sample. The antibody levels between sera at different time points were compared using the two-tailed Wilcoxon matched-pairs ranked sign test. To compare the prevalence of antibody inhibition between different groups of subjects, the two-tailed Mann-Whitney test was used.

#### ELISA for affinity measurement

A relative affinity assay (potassium thiocyanate inhibition) was performed according to a technique described previously [18]. Briefly, microtiter plates were coated and blocked as above. Mabs were added and incubated at 37°C for two hours. After washing, graded concentrations of potassium thiocyanate (KSCN) were added (100 μL/well) in 0.25 mol/L increments with an endpoint concentration of 6 mol/L and incubated for 15 mins at room temperature. All subsequent steps were performed as described for the indirect ELISA. The raw data was plotted to demonstrate a linear relationship between KSCN concentration and antibody-antigen interaction. The molarity of KSCN that would produce a 50% decrease in the calculated maximum OD provides a value for the relative antibody affinity. The greater the molarity required, the greater the relative affinity.

#### Parasite growth inhibition assay

The *in vitro *growth inhibition of *P. falciparum *was assayed as previously described [19] with modifications. Parasites were synchronized by sorbitol lysis twice at a four hour interval to obtain ring stages and allowed to mature to the late trophozoite/schizont stage in the next 24 hours. Synchronously growing parasites were diluted with fresh red blood cells (RBCs) and cultured in 96-well plates at 1% parasitaemia and 1% haematocrit in a total volume of 100 μL, in the presence of anti-MSP4 polyclonal rabbit serum or mouse-derived Mabs. Each serum or Mab sample was tested in triplicate at a final concentration of 20-30% and 250 μg/mL respectively. All sera were heat inactivated at 56°C for 30 mins prior to testing to destroy complement activity, then pre-adsorbed against uninfected RBC (O^+^) to remove any anti-human RBC antibodies. Pre-immune serum was included as a background control. After culture for 40-44 hours, mature-stage parasites were harvested, fixed with 0.025% glutaraldehyde and stained with 10 μg/mL propidium iodide (PI). Fifty thousand cells from each well were analysed by flow cytometry (Becton Dickinson) using 488 nm laser for excitation and 670 nm for emission. Uninfected RBCs stained with PI were used as a negative control and the cut-off was set at 0.05%. The parasitaemia of each sample was calculated using CXP analysis software (Becton Dickinson). The percentage of parasite growth inhibition was calculated according to the equation GI (%) = [(P_C _- P_T_)/ P_C_] × 100, where GI = growth inhibition (%); P_C _= parasitaemia in control and P_T _= parasitaemia in presence of test antibody (rabbit polyclonal sera or Mab). Differences between the test and control cultures were assessed using the two-tailed student *t *test.

## Results

### Generation and characterization of anti-MSP4 Mabs

A panel of nine MSP4-specific Mabs was generated and characterized (Table 1). The specificity of the Mabs was confirmed by ELISA against rMSP4. Positive recognition of trophozoite and schizont stages of *P. falciparum *using indirect IFA further supported the specificity of the Mabs. In addition, all nine Mabs recognized a protein resolving at approximately 40 kDa when immunoblotted against blood stage *P. falciparum *lysates consistent with that previously observed for MSP4. The sequence of MSP4 suggests that the theoretical molecular weight of this protein is 27 kDa, but has previously been reported to show aberrantly slow electrophoretic migration, resolving at 40-45 kDa by SDS-PAGE [2]. The antibody isotype of all nine Mabs was IgG_1 _as determined by an isotype specific ELISA.

**Table 1:** Properties of MSP4-specific Mabs

Mab	Isotype*^a^*	Recognition*^b^*		Reduction	Relative
				
		Fragment	Peptide	Sensitivity*^c^*	Affinity^d^
H12	IgG1	rMSP4A	MSP4.2	*	ND
1B7	IgG1	rMSP4B	none	-	2.2
6G10	IgG1	rMSP4C	MSP4.13	-	2.1
4C10	IgG1	rMSP4D	MSP4.20	**+**	1.7
1G10	IgG1	rMSP4D	MSP4.20	**+**	1.7
7F4	IgG1	rMSP4D	none	**+**	1.4
8G3	IgG1	rMSP4D	none	**+**	1.4
1H2	IgG1	rMSP4D	MSP4.20	-	1.1
6G11	IgG1	rMSP4D	MSP4.20	-	1.6

*a*) Antibody isotype by ELISA. *b*) Fragment specificity by immunoblotting and ELISA and peptide recognition by ELISA. *c*) Reduction sensitivity measured as lack of reactivity (+) to reduced or reduced and alkylated MSP4. *mAb H12 only recognized reduced MSP4. *d*) Antibody affinity determined by ability of thiocyanate to interfere with antigen-antibody binding. ND: not determined.

Initial epitope recognition was carried out by immunoblotting each Mab against the recombinant MSP4 fragment proteins rMSP4A, rMSP4B, rMSP4C and rMSP4D (Table 1). Each fragment corresponds to approximately one quarter of the mature MSP4 protein. Mab H12 recognized the N-terminal quarter of the protein represented by fragment rMSP4A, Mab 1B7 bound to rMSP4B and Mab 6G10 bound to rMSP4C, both representing the central region of MSP4. The remaining six Mabs 4C10, 1G10, 8G3, 1H2, 7F4, and 6G11 bound to rMSP4D, indicating recognition of epitopes in the C-terminal region of MSP4 which includes the EGF-like domain.

**Figure 2:**
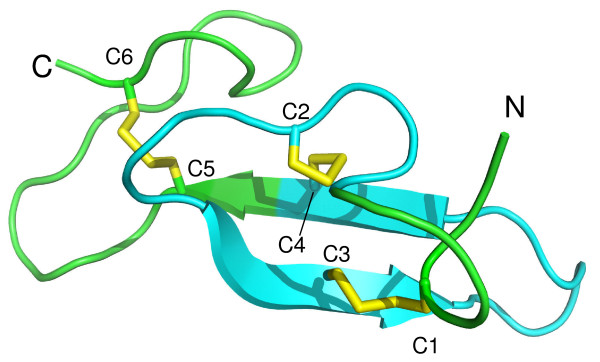
**Cartoon of the structure of the EGF domain from *Plasmodium vivax *Merozoite Surface Protein-1**. Disulphide bonds are highlighted in yellow. The sequence analogous to peptide MSP4.20 (alignment performed using BLAST) in the MSP4 EGF domain is highlighted in cyan. The figure was prepared by James Whisstock using PYMOL (DeLanoScientific LLC.)

The reduction sensitivity of epitopes was examined in more detail by reacting each of the Mabs against rMSP4 that was either non-reduced or reduced and alkylated prior to SDS-PAGE. This treatment destroys conformational epitopes that require disulfide bonds for their integrity. The MSP4D region contains three such bonds (Figure 2). The binding abilities of Mabs 1B7 and 6G10, which recognize the central regions of MSP4, were not affected by the loss of conformational epitopes suggesting that these Mabs recognize linear epitopes of MSP4. Interestingly, Mab H12 only recognized reduced rMSP4, suggesting that the original rMSP4 immunogen may have been partially denatured. The epitopes of the C-terminal region were examined by testing each of the rMSP4D-positive Mabs against both rMSP4 and rMSP4D as non-reduced or reduced and alkylated proteins. Binding of Mabs 4C10, 1G10, 7F4 and 8G3 was abolished when reacted with reduced and alkylated proteins. The same results were observed with these Mabs when reacted with either reduced or reduced and alkylated parasite lysates. These observations indicate that these Mabs are directed to conformational epitopes that are dependent on disulphide bond formation. The binding abilities of Mabs 6G11 and 1H2 were not affected by reduction of the target epitopes indicating that they recognize linear epitopes within the C-terminus of MSP4.

The affinity of the Mabs was determined by the ability of thiocyanate to interfere with antigen-antibody binding in a concentration dependent manner, and are shown in Table 1. The relative affinity of the Mabs ranged from 1.1 (1H2) to 2.2 (1B7).

### Epitope mapping of anti-MSP4 Mabs

To further define the epitopes of the C-terminal region of MSP4, the ability of the six anti-MSP4D Mabs to block each other's binding to rMSP4D was assessed using competition ELISA (Table 2). Binding of Mab 4C10 and 1G10 to rMSP4D were completely blocked by each other. Similarly, Mabs 7F4 and 8G3 showed competitive binding. This indicates that the 4C10/1G10 and 8G3/7F4 Mab pairs recognize identical or highly overlapping epitopes. Mab 1H2 competed with Mabs 4C10/1G10 and 6G11 for binding to rMSP4D, but not with 8G3/7F4, whereas Mabs 4C10/1G10 were shown to compete with 6G11. The epitopes for Mabs 1H2, 4C10/1G10 and 6G11 are therefore likely to overlap with each other but individually they are distinct as 6G11 and 1H2 are directed to linear epitopes whereas 4C10 and 1G10 recognize a conformational epitope (Table 1). Mabs 8G3/7F4 recognize a separate, distinct conformational epitope.

**Table 2:** Mapping of Overlapping Epitopes of the MSP4 EGF-like Domain

Competitor	Competition for Binding with Biotinylated Mab					
	
Mab	4C10	1G10	7F4	8G3	1H2	6G11
4C10	**+**	+	-	-	-	+
1G10	+	**+**	-	-	-	+
7F4	-	-	**+**	+	-	-
8G3	-	-	+	**+**	-	-
1H2	+	+	-	-	**+**	+
6G11	-	-	-	-	-	**+**

Mabs specific for rMSP4D analysed by pairwise competition ELISA.

Inhibition (+) or no inhibition (-) of binding was observed.

Overlapping synthetic peptides spanning the entire length of the MSP4 molecule were used in an ELISA to further map Mab recognition sites to 20-amino acid linear epitopes (Figure 1). Mab H12, which only recognized reduced rMSP4 and rMSP4A, mapped to the N-terminal peptide MSP4.2. Mab 1B7, which recognizes a reduction-insensitive epitope within rMSP4B did not bind to any of the peptides. Mab 6G10, which targets a reduction-insensitive epitope in rMSP4C, was shown to bind to peptide MSP4.13. This peptide spans the junction of the MSP4B and MSP4C regions with seven residues in the B region and thirteen residues in the C region. As this Mab recognized rMSP4C but not rMSP4B it was possible to further map this epitope to the thirteen residue sequence AIPKKVVQPSSSN (underlined in Figure 1) which lies within the MSP4C region. Epitope mapping using peptides spanning the MSP4D region containing the EGF-like domain was also performed. Mabs 7F4 and 8G3 did not show detectable binding to any of the synthetic peptides, consistent with the conformational nature of the epitope they recognize. Mabs 1H2 and 6G11, which recognize reduction-insensitive linear epitopes, bound to peptide MSP4.20. Surprisingly, Mabs 4C10 and 1G10, which recognize reduction-sensitive epitopes, were also able to bind to peptide MSP4.20. This suggests that their epitopes are either partially linear, or that two of the three cysteines within this peptide are producing a conformation that at least partly forms the epitope for 4C10 and 1G10. The 3D structure of the EGF-like domain of some *Plasmodium *merozoite surface proteins, such as that of *Plasmodium vivax *Merozoite Surface Protein 1 (PvMSP1) have been resolved [20]. According to the published 3D structure of PvMSP1, the pairing of the disulphide bonds occurs between C1-C3, C2-C4, and C5-C6 (Figure 2). BLAST was used to align the predicted location of the sequence analogous to peptide MSP4.20 in the PvMSP1 EGF domain and is shown in Figure 2. Peptide MSP4.20 contains cysteines C2, C3 and C4 and therefore this area is involved in the formation of two disulphide bonds, being C1-C3 and C2-C4. It is concluded that the conformational epitope recognized by Mabs 4C10 and 1G10 is likely to involve the disulphide bond formed between C2 and C4.

### Naturally acquired human antibodies compete with anti-MSP4 Mabs

It was of interest to determine whether antibodies induced during natural infection of humans recognized similar epitopes to those seen by mice immunized with recombinant MSP4. Human sera were sampled from individuals who had demonstrable *P. falciparum *parasitaemia. Sera were collected at three different time points: T_0 _at the beginning of the survey and prior to drug treatment, T_1 _at the time of re-infection and drug treatment and T_28 _collected from the same individuals 28 days later. The four regions of MSP4 represented by rMSP4A, rMSP4B, rMSP4C and rMSP4D were recognized by the sera from these individuals suggesting that the human immune response to MSP4 is directed to at least four distinct epitopes [6,7].

**Figure 3:**
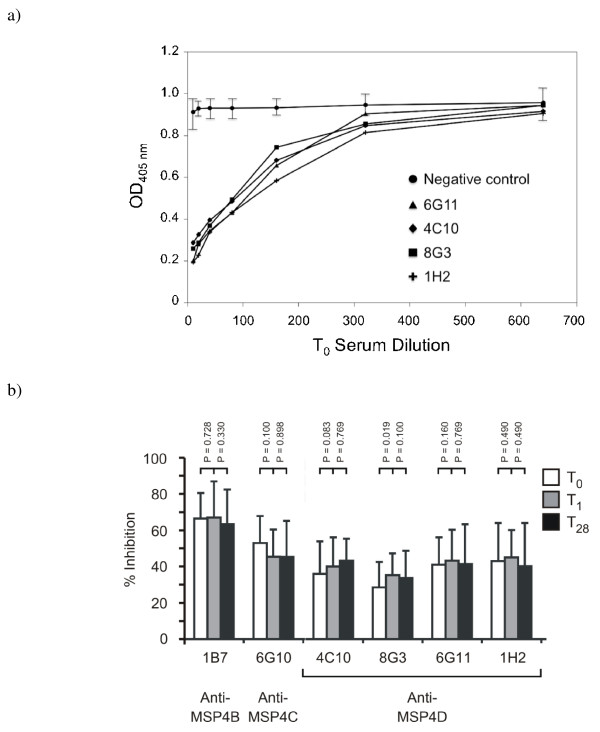
**Competition of naturally acquired human antibodies with anti-MSP4 specific Mabs**. Serum samples were collected from individuals who acquired and were treated for *P. falciparum *parasitaemia at different time points; T_0_, at the time of infection; T_1 _and T_28_, day 1 and day 28 following infection. a) Inhibition of Mab binding to MSP4 by a pool of human sera from infected individuals. A pool of human sera collected at the time of infection (T_0_) were reacted with MSP4 by ELISA followed by addition of four Mabs (specific for the EGF like domain of MSP4) to determine the ability of human sera to inhibit the binding of Mabs. The human serum pool was able to inhibit Mab binding in a dose-dependent manner. The serum dilution is shown on the X axis and Absorbance value is shown on the Y axis. b) Heterogeneity of the human MSP4-specific antibody response. Human sera collected at different time points (T_0_, T_1 and _T_28_) were competed with Mabs raised to different regions of MSP4 in the competition ELISA assay. Bars indicate the average percentage inhibition (%) at 1/10 dilution of 10 samples of human sera tested at each timepoint and the error bars indicate standard deviation within the sample. The *P *values (Wilcoxon test) between the inhibition in the matched pairs of serum samples at the different timepoints are shown.

The ability of naturally acquired antibodies in human immune sera to block the binding of selected MSP4 Mabs was tested in a competition ELISA. A total of 84 immune sera were examined. Initially, Mabs directed against conformational (4C10 and 8G3) and linear (1H2 and 6G11) epitopes within rMSP4D were tested in the presence of human serum collected at the beginning of the survey (T_0_). A pool of human sera were able to block the binding of all four Mabs in a dose-dependent manner, indicating that C-terminal conformational and linear MSP4 epitopes are recognized by both the Mabs and the human immune sera (Figure 3a). The data shows that there is considerable variation between individuals in the ability of their serum antibodies to block the binding of Mabs to MSP4 (Figure 3b). The IgG responses at the beginning of the survey (T_0_), during infection (T_1_) and convalescent phase (T_28_) of infection were examined in competition with Mabs recognizing epitopes within the central (MSP4C/D) and C-terminal (MSP4D) regions of MSP4 (Figure 3b). IgG responses at each time point were directed towards all three of the regions tested. Serum titres varied between 1/20 and 1/640, showing considerable variation between individuals. The average percentage inhibition of sera from each patient remained relatively constant before and during infection and through convalescence.

### Polyclonal anti-MSP4 antibodies inhibit *in vitro *parasite growth

Rabbit polyclonal antisera raised against full-length rMSP4 inhibit parasite growth *in vitro *to a moderate but significant level (Table 3). Of the five antisera generated against rMSP4, three showed growth inhibition that was above 40%. These antisera also showed higher anti-rMSP4 titres with S1619 at 1 × 10^5^, whereas S1621 and S1622 (the antisera raised against *S. cerevisiae*-expressed protein) showed a titre of over 1 × 10^6^. The other two antisera raised against full length rMSP4 (S193 and S1620) had an antibody titre of 3 × 10^4 ^and showed no detectable growth inhibition. These results suggest a positive correlation between the *in vitro *growth inhibitory capacity of antisera and antibody titre. The growth inhibitory capacity of antisera raised against fragments of the MSP4 molecule was also examined. In contrast to full-length protein, antisera raised against fragments rMSP4A, rMSP4B, rMSP4C and rMSP4D gave negligible parasite growth inhibition *in vitro*. This suggests that multiple MSP4 epitopes spanning the entire protein need to be targeted in order to significantly inhibit parasite growth *in vitro*. These results were consistent with those obtained using individual anti-MSP4 Mabs to inhibit parasite growth. The extent of growth inhibition obtained with the purified Mabs was not significant. To determine if three antibody specificities in three different fragments would be able to demonstrate inhibition, Mabs 6G10 (anti-MSP4C), 1B7 (anti-MSP4B) and 4C10 (anti-MSP4D) were tested in combination. Significant growth inhibition was not obtained by this combination of antibodies. This suggests that antibodies directed to more than such three epitopes spread across the molecule are necessary to result in parasite growth inhibition.

**Table 3:** *In vitro *Growth-Inhibitory Activities of MSP4-Specific Antibodies

Antibody Reagent	Immunogen^1^	Specificity^2^	MSP4 Titre^3^	% GIA^4^
*Polyclonal Sera*				
S193	rMSP4^GST^	Full MSP4	30,000	12
S1619	rMSP4	Full MSP4	100,000	47*
S1620	rMSP4	Full MSP4	30,000	15*
S1621	rMSP4^Sc^	Full MSP4	1,000,000	41**
S1622	rMSP4^Sc^	Full MSP4	1,000,000	64**
S104	rMSP4A	MSP4A	ND	14
S127/162	rMSP4C	MSP4C	ND	-26
S133	rMSP4D	MSP4D	ND	-18

*Mabs*				
H12	rMSP4	MSP4A		6
1B7	rMSP4	MSP4B		13
6G10	rMSP4	MSP4C		12
4C10	rMSP4	MSP4D		10
1G10	rMSP4	MSP4D		8
7F4	rMSP4	MSP4D		8
8G3	rMSP4	MSP4D		7
1H2	rMSP4	MSP4D		5
6G11	rMSP4	MSP4D		9
6G10+1B7+4C10	rMSP4	MSP4B/C/D		11
PBS control	-	-		5

*Control Reagents*				
Anti-MSP1_19 _sera				51**
Anti-AMA-1 purified IgG				88**
Anti-GST sera				10

1) Polyclonal and monoclonal antibodies were raised against recombinant MSP4 produced in *E.coli *as a hexahistidine (rMSP4) or GST (rMSP4^GST^, rMSP4A/C/D) tagged protein. S1621 and S1622 were raised against *S. cerevisiae*-expressed protein (rMSP4^Sc^). 2) Specificity confirmed by ELISA and/or immunoblotting. 3) Titre for selected polyclonal sera determined by ELISA against rMSP4. 4) Parasite growth inhibition assay (GIA) results (%). Statistical significance of inhibition by two-tailed student *t*-test comparing final parasitaemias in wells with test antibody to control culture; p < 0.1 (*), p < 0.01 (**) and p > 0.1. ND: not determined.

## Discussion

The natural immune response to malaria is highly complex involving both antibodies and cell mediated immunity [21]. The clinical symptoms of malaria are caused by the asexual blood stage. Antibodies are particularly important in protective immunity during this stage as they can bind to antigens on free merozoites and inhibit erythrocyte invasion either directly or in association with cells to limit disease pathogenesis and clinical symptoms. Evidence for the protective role of antibodies in clinical malaria are provided from studies where passive transfer of antibodies from immune adults was able to successfully treat children with severe *P. falciparum *infection [22]. However, the finding that long-term exposure to the parasite resulting in multiple antibody specificities is necessary to generate protection from malaria indicates that the establishment of protective antibodies is a complex process. The breadth and magnitude of the antibody responses to multiple merozoite antigens are shown to be associated with protection from clinical malaria [23]. The antibody response to merozoite surface protein 1 (MSP1) has been extensively studied and particular areas of the protein have been found to be immuno-dominant [17]. For example, antibodies against the 19 kDa C-terminal fragment of MSP1 are the major component of the invasion inhibitory response in humans immune to malaria [19,24]. The protective effect of these anti-MSP1_19 _antibodies is further dependent on their fine specificity, rather than mere prevalence or titre [25]. Similar studies suggest the existence of immune-dominant functional domains that are the target of protective antibodies to apical membrane antigen-1 (AMA-1) [26,27]. In contrast, our previous immuno-epidemiological studies showing recognition along the length of MSP4 [6], as well as the fine mapping and functional studies presented herein, suggest the possibility that acquisition of protective antibody immunity to MSP4 could be a cumulative process with a requirement for the generation of multiple antibody specificities.

In this study, nine monoclonal antibodies specific for recombinant MSP4 protein expressed in *Escherichia coli *were produced and characterized. These Mabs recognize six distinct epitopes of MSP4, including two conformational epitopes in the C-terminal region containing the EGF-like domain. The remaining Mabs recognize epitopes that are not affected by reduction and alkylation and are presumably linear, with two in the C-terminal region of MSP4 and two in the central region of the protein. The reactivity of Mab H12 was interesting in that it reacted only with the reduced and alkylated form of full-length MSP4 but mapped to a region of the molecule, MSP4A, that has no disulphide bonds. This suggests that there are long range conformational effects which alter the shape of the N-terminus of MSP4 when disulphide bonds in the C-terminus are interrupted. This is in accord with previous observations in which the redox state of the EGF-domain was crucial for the antigenicity of the entire protein, including regions that are not immediately adjacent in the primary structure and did not contain disulphide domains such as the N-terminus [5].

This panel of antibodies was used in competition ELISA to analyse the binding specificities of naturally acquired antibodies to MSP4 in individuals from a malaria-endemic region of Vietnam. The three tested regions of MSP4 were readily recognized by the sera examined. The existence of persistent heterogeneity was observed among individuals in the level of antibody reactivity and the spread of epitope recognition. The reactivity of anti-MSP4 antibodies in the sera tested was considerably higher for epitopes in the central region of MSP4, in particular the MSP4B region. This result is also in agreement with our previous findings [6] that the percentage of positive responses was higher for MSP4B (92.5%) compared to the EGF-like domain containing MSP4D region (71.3%). This is also in accord with other reports where cysteine-rich regions of proteins have been shown to be less immunogenic [28]. In contrast, six of the nine Mabs generated in this study by immunization of mice with rMSP4 were specific for the EGF-like domain.

MSP4 is a surface exposed protein that is highly conserved. The finding that antibodies target multiple epitopes spread across the entire length of the molecule may provide some insight into the mechanisms for co-evolution of parasite with its human host. It is possible to speculate that the sequence conservation of MSP4 maybe partly caused by the fact that immune selective pressure needs to target multiple epitopes simultaneously rather than individual epitopes. In addition to helping in understanding the nature of the antibody response to MSP4 during infection, the panel of monoclonal antibodies will also serve as valuable reagents for testing product integrity, purity, antigenicity and immunological activity during the highly complex and stringently regulated process of MSP4 vaccine development and manufacture.

It is shown that polyclonal rabbit antisera raised against the full length MSP4 protein are able to inhibit parasite growth *in vitro*. In contrast, rabbit antisera specific for MSP4 fragments corresponding to three of the regions of MSP4 were unable to inhibit parasite growth. Similarly, the individual Mabs had no inhibitory effect on the growth of the parasite. These results are consistent with those previously obtained using an *in vivo P. yoelii *murine malaria challenge model in our laboratory. Immunizations carried out with recombinant PyMSP4/5 were able to induce immune responses that protect mice against challenge with a lethal dose of *P. yoelii *parasites [10]. In contrast, immunizations with PyMSP4/5 fragments did not confer protection (L. Kedzierski, unpublished data). One explanation for this is that an antibody response directed to multiple epitopes spread across the molecule is necessary for arresting parasite growth. Since the combination of three Mabs directed to different regions of the MSP4 molecule were also unable to produce a growth inhibitory effect, it is likely that a polyclonal antibody response to more than three epitopes is necessary. Alternatively another possibility is that antibodies directed to epitopes other that those represented by the Mabs are required for invasion inhibition. These epitopes maybe ones that are only present in the complete folded sequence and accessible on the merozoite surface. The lack of inhibitory activity despite the high affinity binding of the Mabs may also be related to the function and size of the MSP4 molecule. In the case of AMA-1, inhibitory antibodies bind and block epitopes that are involved in the parasite invasion process. MSP4 is a small molecule (270 amino acids) and of relatively low abundance on the surface of the merozoite. Hence, the binding of antibodies to MSP4 alone may not be sufficient to cause high levels of parasite dysfunction or invasion inhibition, but MSP4-specific antibodies may contribute in combination with antibodies specific for other merozoite surface proteins.

Following schizont rupture and merozoite release, re-invasion occurs within seconds. Therefore, antibodies will only be useful if they are available at very high concentrations and/or are of high affinity. Consistent with this picture, the observations in this study showed that growth inhibitory capacity is directly proportional to antibody titre. Similar observations have been made in field studies in malaria endemic areas; under natural exposure, immunity to malaria results from high titre antibodies to multiple antigenic targets [23]. These observations provide support for the production of combination blood stage vaccines.

The capacity to induce experimental antibodies with growth inhibitory activity (GIA) *in vitro *is currently being used as a criterion for selection of blood stage antigens that are to be incorporated into a subunit malaria vaccine. Growth inhibition *in vitro *is considered a functional assay because it measures the capacity of antibodies to bind and inhibit parasite invasion and/or growth. AMA-1 [27] and MSP1 [29] are the blood stage antigens currently in the most advanced stages of being developed as human malaria vaccines. Antibodies to AMA-1 so far have been able to show consistently high levels (> 80%) of growth inhibition *in vitro *[30,31] and much attention has focused on this protein as a promising vaccine candidate against malaria. In contrast, anti-MSP1_19 _antibodies show more moderate inhibitory activity [29,32,33]. If growth inhibitory activity is sufficient for protection, then an effective malaria blood-stage vaccine would likely need to be made up of multiple antigens. Growth inhibition leading to decreased replication through the erythrocytic cycle is likely to be a result of the collective immunogenic ability of the multiple antigens. On this basis a growth inhibitory capacity of 40% observed for MSP4 as a single antigen would be a significant contribution. Further studies are required to determine if the level of growth inhibition is increased when antibodies directed to several merozoite surface antigens are tested in combination.

Despite extensive work the value of using GIA as the sole correlate of immune protection from *Plasmodium *infections is still unclear. The validity of this *in vitro *assay may be confirmed once vaccine trials are carried out in humans and *in vitro *inhibition is shown to correlate with *in vivo *protection. A recent Phase IIa trial of AMA-1 has shown disappointing results in this regard [34]. Antibodies classically act in conjunction with other mechanisms of immunity, such as complement mediated destruction of the pathogen and enhanced phagocytic activity mediated by antibody opsonization. These mechanisms are also likely to be operating in immunity to blood stage malaria. Interestingly, the majority of human antibodies recognizing MSP4 were of the IgG3 and IgG1 isotypes suggesting that they may play a role in opsonization and complement-mediated destruction of free merozoites [6].

The Antibody Dependent Cellular Inhibition or Cytotoxicity assay (ADCI or ADCC) is another *in vitro *assay that can be used to determine antibody efficacy in co-operation with monocytes to limit *P. falciparum *growth [35,36]. Interestingly, a positive ADCI has been demonstrated for other blood stage antigens MSP3 [37] and GLURP [38], despite the lack of *in vitro *growth inhibition by antibodies alone. In view of this finding, our antisera raised against MSP4 are currently being tested for ADCI activity.

Apart from antibody-mediated immunity, cellular mechanisms such as the production of IFNγ-producing T cells are also important in mediating protection against blood stage malaria [39,40]. T cells that recognize dominant epitopes in vaccine proteins are essential for long-term protection against malaria in animal models. Preliminary work was carried out to predict the possible T cell epitopes of MSP4 in humans that bind across diverse HLA molecules using an epitope prediction algorithm [41]. All four regions of the molecule contained areas that were predicted to have T cell epitopes and these areas also overlapped with the three peptides containing B cell epitopes. These findings have important implications for vaccine design enabling incorporation of peptide antigens that are able to stimulate strong T and B cell responses to MSP4.

## Conclusions

A panel of Mabs specific for *P. falciparum *MSP4 were generated, characterized and used to develop an MSP4-specific competition ELISA to test antibody specificity in sera from naturally infected individuals. All epitopes recognized by the Mabs were readily recognized by human immune sera, with heterogeneity in the intensity of the humoral response among different individuals. Growth inhibition assays were carried out comparing growth inhibitory activity of the Mabs and polyclonal rabbit sera. The results demonstrate that multiple MSP4 epitopes spanning the entire protein need to be targeted to significantly inhibit *P. falciparum *growth. These findings provide insight into the MSP4 specific antibody response and have important implications for the design of blood stage vaccines.

## Competing interests

The authors declare that they have no competing interests.

## Authors' contributions

RLC conceived the study. HdeS and SS designed and performed the experiments and analysed the data. HdeS wrote the manuscript. MP and SK helped with revision of manuscript. LW, CB and MP contributed information and reagents. All authors read and approved the final manuscript.
